# Feasibility, acceptability, and preliminary effects of the Join4Joy approach for older adults living in nursing homes: a multicentre mixed-methods feasibility study

**DOI:** 10.1007/s41999-026-01547-2

**Published:** 2026-08-31

**Authors:** Erika Karkauskiene, Laura Coll-Planas, Andrea Fuente-Vidal, Damaso Sanchez, Nadine Barth, Dhayana Dallmeier, José Luis Socorro-Cumplido, Maria Giné-Garriga, Paolo Caserotti, Guillaume Lefebvre, Javier Jerez-Roig

**Affiliations:** 1 Department of Health Promotion and Rehabilitation, Lithuanian Sports University, Kaunas, Lithuania; 2 Research Group on Methodology, Methods, Models and Outcomes of Health and Social Sciences (M3O), Faculty of Health Sciences and Welfare, Centre for Health and Social Care Research (CESS), University of Vic-Central University of Catalonia (UVic-UCC), 08500 Vic, Spain; 3 Institute for Research and Innovation in Life Sciences and Health in Central Catalonia (IRIS-CC), Vic, Spain; 4 Institute for Geriatric Research, Ulm University Medical Center, Ulm, Germany; 5 Agaplesion Bethesda Clinic Ulm, Ulm, Germany; 6 Department of Epidemiology, Boston University School of Public Health, Boston, USA; 7 Department of Physical Activity and Sport Sciences, School of Psychology, Education and Sport Sciences (FPCEE) Blanquerna, Universitat Ramon Llull, Barcelona, Spain; 8 Department of Physiotherapy, School of Health Sciences (FCS) Blanquerna, Universitat Ramon Llull, Barcelona, Spain; 9 Department of Sports Science and Clinical Biomechanics, Center for Active and Healthy Ageing, University of Southern Denmark, Odense, Denmark; 10 Department of Sports Science and Clinical Biomechanics, Center for Active and Healthy Ageing (CAHA), University of Southern Denmark, Odense, Denmark; 11 Sport Initiative et Loisir Bleu (SIEL BLEU), Strasbourg, France; 12 Institute of Sport Science and Innovations, Lithuanian Sports University, Kaunas, Lithuania

**Keywords:** Nursing home, Physical activity, Frailty, Feasibility study, Older adult

## Abstract

**Purpose:**

Nursing home residents increasingly enter care with advanced frailty, multimorbidity, and functional dependence, underscoring the need for engaging, adaptable physical activity (PA) programmes. Join4Joy is a co-created approach designed to promote movement and social connection among older adults through enjoyable activities. This study examined its feasibility, acceptability, and preliminary effects across five nursing homes in Spain, France, and Germany.

**Methods:**

This multicentre mixed-method study (ClinicalTrials.gov: NCT06100835) was delivered in two phases. Residents aged ≥ 65 years participated in 12 weekly 60-min PA sessions. Feasibility was assessed through attendance, procedural indicators, data completion, and retention; acceptability was evaluated using participant satisfaction and qualitative feedback, while safety was monitored throughout. Quantitative assessments evaluated physical function (SPPB), daily functioning (modified Barthel Index), quality of life (EQ-5D-5L), PA and sedentary behaviour (IPAQ-SF, SBQ, accelerometry), perceived improvement (PGI-I), and enjoyment (PACES-SF). Qualitative interviews and focus groups explored participant experiences.

**Results:**

Of 81 enrolled residents, 75 completed the intervention (retention = 92.6%). Attendance was high (73.8–95.0%), no adverse events occurred. Quantitative outcomes indicated small favourable or no changes in physical and daily functioning, quality of life, PA and sedentary behaviour, perceived improvement, and enjoyment. Qualitative themes highlighted enjoyment, social connection, confidence, and the value of tailored, playful activities, confirming high acceptability.

**Conclusion:**

Join4Joy is safe, feasible to deliver, and well received, demonstrating the viability of a future definitive trial. Future large-scale evaluations should integrate holistic psychosocial metrics like enjoyment and social connection alongside biomedical outcomes to fully assess co-created PA approaches in long-term care geriatric settings.

**Supplementary Information:**

The online version contains supplementary material available at https://doi.org/10.1007/s41999-026-01547-2.

## Key summary points

**Aim:**

To examine the feasibility, acceptability, and preliminary effects of the co-created Join4Joy approach in nursing home residents, across three European countries.

**Findings:**

The intervention was feasible, safe, and well accepted with strong satisfaction, and no reported adverse events. Quantitative outcomes showed small positive changes or maintenance in physical and psychosocial measures, while qualitative data highlighted enjoyment, social connection, and perceived functional benefits.

**Message:**

Join4Joy presents a co-created and enjoyable physical activity-based approach that may support engagement, well-being, and functional maintenance among nursing home residents.

**Supplementary Information:**

The online version contains supplementary material available at https://doi.org/10.1007/s41999-026-01547-2.

## Introduction

Population ageing is accelerating worldwide, leading to a growing number of older adults requiring long-term care. Evidence shows that today’s nursing home (NH) residents enter institutional care with more advanced frailty, multimorbidity, and functional limitations than previous generations, reflecting increasing complexity of care needs [1–4]. Consequently, frailty, sarcopenia, and functional dependence have become highly prevalent and represent major clinical challenges within NHs. Frailty, cognitive decline, and disability are highly prevalent among NH residents [5–7]. Physical inactivity and sedentary behaviour accelerate the biological processes underlying frailty—such as declines in muscle mass, neuromuscular coordination, and cardiovascular capacity, thereby increasing the risk of falls, disability, and hospitalization [8]. As a result, maintaining functional capacity and quality of life remains a key priority for institutionalised older adults [9].

Regular physical activity (PA) is recognised as a non-pharmacological strategy that may support functional capacity and overall health in older adults [9]. Systematic reviews suggest that multicomponent exercise programmes may improve functional performance and mobility among older adults [4, 10, 11]. Structured, group-based multicomponent exercise programmes, even when adapted to frail and cognitively impaired older adults, have been associated with improvements in their physical and mental health outcomes [12]. Evidence suggests that even in very late life, appropriately adapted PA carries low risk and may support functional maintenance, symptom control, and quality of life, rather than accelerating decline [13, 14]. However, despite this evidence, the implementation of structured exercise programmes in real-world NH practice remains challenging [15]. Furthermore, when implemented, long-term effectiveness is often constrained by low adherence and limited sustainability in NH settings [16].

Barriers include organisational constraints [16], limited staff resources [16, 17], heterogeneity in residents’ physical and cognitive abilities [16, 18], and low motivation to engage in the conventional exercise routines [18]. Interventions that are not tailored to individual preferences or that neglect social and emotional engagement often experience poor adherence [19]. Consequently, there is a growing interest in developing PA programmes that are enjoyable, adaptable and co-created with end-users: integrating movement into everyday institutional life while promoting social connections and psychological well-being [20].

The Join4Joy project was developed to promote PA and social connection in NH residents through a co-created, enjoyable, and adaptable approach [21]. Grounded in the Octalysis gamification framework [22], it emphasises social inclusion, engagement, and flexibility [23]. The approach integrates multicomponent movement with playful and socially interactive activities, reflecting a biopsychosocial model of ageing [24].

Although previous studies have shown that multicomponent exercise programmes can enhance mobility and independence in NH residents [25, 26], it remains unclear whether programmes designed around enjoyment and social inclusion can achieve improvements in physical function and well-being within frail institutionalised populations. Therefore, our mixed-method feasibility study aimed to evaluate the feasibility, acceptability, and preliminary biopsychosocial effects of the Join4Joy approach among NH residents across three European countries. Feasibility was assessed through attendance and retention across two phases, while acceptability included satisfaction and perceived experiences. In addition, pre–post-changes in physical function, daily functioning, quality of life, PA, sedentary behaviour, perceived improvement, and enjoyment were also explored as secondary outcomes to provide preliminary indicators of potential biopsychosocial benefits.

## Methods

### Ethical considerations

The study was conducted in accordance with the ethical principles of the Declaration of Helsinki and was registered at ClinicalTrials.gov (NCT06100835). Ethical approval for the Join4Joy project was obtained from the Research Ethics Committee of the University of Vic—Central University of Catalonia (UVic-UCC). Approval for the co-creation phase was granted on 3 October 2022 (internal code 233/2022), and for the intervention protocol on 26 June 2023 (internal code 282/2023), covering both the Spanish and French sites. In Germany, additional approval was provided by the Ethics Committee of the University of Ulm on 31 October 2023 (internal code 297/23). All data of this study were coded using participant identification numbers to ensure confidentiality.

### Study design

This study adhered to the published Join4Joy protocol [21], which explains its theoretical basis, co-creation process, and implementation structure. It follows the CONSORT 2010 guideline extension for pilot and feasibility trials [27]. The study followed a multicentre, mixed-methods feasibility design with a non-randomised, single-group, pre-post structure, conducted in two sequential phases. In Phase 1 (November 2023–April 2024), we conducted initial delivery, followed by reflection, sharing and refinement of procedures, as required, and in Phase 2 (July 2024–February 2025), we delivered based on Phase 1 learnings.

Both phases followed the same overall protocol and assessment framework, while Phase 2 incorporated procedural refinements informed by the delivery experience of Phase 1. All quantitative data were pooled for final analysis. The study followed a four-phase framework. First, a co-creation phase involving older adults, their relatives, care assistants, higher education students, policy-makers, and specialists shaped the conceptual basis of the approach. Second, facilitators and collaborating students completed online training to standardise the delivery process. Third, the intervention was delivered across two phases in NH settings. Finally, qualitative and quantitative data were analysed to assess the outcomes. As a feasibility study, with a focus on implementation, acceptability, and process outcomes in a real-world NH settings, a control group was not included at this stage.

### Participants

#### Eligibility criteria

The Join4Joy project prioritised inclusivity and diversity, aiming to engage NH residents who typically face higher barriers to participation in PA programmes. The inclusion approach was designed to reflect real-world conditions while maintaining safety and feasibility. Eligible participants were aged 65 years or older, residing in NHs or attending its day-care services, and able to participate safely in structured, group-based PA sessions. Day-care attendees were included, because they shared the same care environment, participated in the same group-based activities, and represented a relevant subgroup for assessing the feasibility and acceptability of the approach in real-world long-term care settings. Of the total sample, 19 participants (25.3%) were recruited from day-care services.

Residents were excluded if they had severe cognitive decline (i.e., stage 7 on the Global Deterioration Scale of  Reisberg), severe mobility limitation (i.e., being bed-bound), or medical conditions contraindicating light-to-moderate activity (e.g., unstable cardiac disease or acute infection). No exclusions were applied based on age, chronic illness, or other sociodemographic characteristics if participation was considered safe by care staff and facilitators.

#### Settings and locations

The Join4Joy approach was delivered in NHs across three European countries representing different long-term care models. NHs were selected using purposive sampling through established project partners across participating countries. In Spain, the intervention was delivered in two NHs: one large (≈ 130 beds) and one smaller mixed residential/day-care centre (≈ 40 residential beds and 15 day-care places). In France, the intervention was applied in two NHs providing daily nursing and rehabilitation-oriented support. In Germany, the intervention was conducted at a medium-sized (≈75 beds) NH that forms part of a broader geriatric care network integrating acute and rehabilitative geriatric services. All facilities signed collaboration agreements before the study conduction. Assessments and intervention sessions were conducted on-site to ensure accessibility, safety, and ecological validity.

#### Recruitment and consent

NH staff screened residents for eligibility, provided initial information sessions, and introduced researchers to potential participants. After detailed oral and written explanations, participants provided written informed consent prior to baseline assessment. When decision-making capacity was lacking, written informed consent was obtained from legal representatives in accordance with local ethical procedures. Participants’ willingness to take part was respected throughout the study and they were informed of their right to discontinue participation at any time. Those who chose to do so, were asked to voluntarily indicate their reasons for withdrawal, allowing the research team to assess intervention’s feasibility.

### Intervention description

The Join4Joy approach emphasised tailoring and adaptability. Prior to delivery, most centres conducted one-on-one interviews with each participant. These interviews explored participants’ previous activity experiences, preferences, perceived limitations, motivations, and safety considerations. Although not used for data analysis, this information guided the tailoring of activities to maximise engagement and safety. The intervention consisted of weekly 60-min sessions delivered over approximately 12 weeks. Sessions incorporated multicomponent exercises, including aerobic activities, strength training, coordination and balance, flexibility, and rhythmic or playful movement tasks, corresponding to light-to-moderate intensity (approximately 3–5 METs). Exercise intensity (light-to-moderate) was monitored by facilitators through observation of visible signs of fatigue or discomfort, consistent with light-to-moderate intensity delivery. No formal perceived exertion or fatigue scale was used. Some of the activities were co-created with residents and facilitators, ensuring adaptability to individual physical and cognitive abilities while aiming to maintain group cohesion.

Facilitators included NH care assistants and professional staff (i.e., physiotherapists, occupational therapists), external professional staff (i.e., sport scientists), and supervised students who had previously undergone the Join4Joy online training. The latter provided practical guidance on approach delivery, enabling staff from diverse professional backgrounds to tailor it to their local context. Throughout the intervention, facilitators were encouraged to promote active lifestyles beyond the structured sessions, reinforcing everyday movement and social participation as part of the Join4Joy philosophy. This typically involved simple homework tasks or recommendations, such as taking more steps during the day, standing or walking during leisure time, or choosing to be more active within their living environment, consistent with the Join4Joy philosophy. Further details on the Join4Joy intervention components are available in the published protocol and publication on co-design [21, 23]. A detailed description of the intervention components, structured according to the Template for Intervention Description and Replication (TIDieR) checklist, is provided as Supplementary Material.

### Outcome measurements

At baseline, sociodemographic and anthropometric information (age, sex, education level, marital status, prior PA experience, comorbidities, height, and weight) was collected to characterise the study population. Feasibility outcomes were measured using recruitment, attendance, and retention rates, as well as successful intervention delivery, research processes, multicentre working, and data completion. Attendance was defined as the proportion of attended sessions relative to the total number of sessions offered during the intervention period. Retention was defined as the proportion of participants who completed the intervention and post-intervention assessments relative to those initially enrolled. Facilitators were encouraged to monitor any adverse events and record them in structured session diaries. Adverse events were defined as any unexpected physical, medical, or safety-related incident occurring during or immediately after the sessions.

Physical outcomes were assessed before and after the intervention using the Short Physical Performance Battery (SPPB) [28] test for physical function and the modified Barthel Index [29] for daily functioning. Quality of life was measured with the EQ-5D-5L index and its visual analogue scale (VAS) [30]. Enjoyment of PA and perceived improvement were assessed post-intervention only by means of the Physical Activity Enjoyment Scale-Short Form (PACES-SF) [31] and the Patient Global Impression of Improvement-I (PGI-I) scale [32].

PA and sedentary behaviour were evaluated both objectively and subjectively. In Spain and Germany, device-based assessment using wrist-worn ActiGraph GT3X + accelerometers (sampling rate 30 Hz, 60-s epochs) was used. Participants were instructed to wear the device on their dominant wrist for seven consecutive days, before and after the intervention. Data were considered valid when the device was worn for at least 10 h per day on ≥ 5 valid days, including at least one weekend day. Raw data were processed using standard ActiLife software to calculate average sedentary time, light activity, and moderate-to-vigorous PA (MVPA). Sedentary behaviour and PA intensity levels (light, and moderate-to-vigorous) were calculated based upon recommended PA vector magnitude cut points: 0-1853, 1854-9267, 9268, and above CPM (counts per minute), respectively [33]. Participants were also provided with accelerometry diaries to record non-wear periods and reasons for not wearing the device. However, these diaries were not included in the analysis due to incomplete and inconsistent reporting. All countries also used the International Physical Activity Questionnaire-Short Form (IPAQ-SF) [34] to subjectively capture walking, moderate and vigorous activity, and sedentary time. The Sedentary Behaviour Questionnaire (SBQ) [35] estimated time spent on various sedentary activities (e.g., watching television, reading, or computer use) on both weekdays and weekends.

Acceptability of the intervention was assessed by measuring satisfaction. It was measured via a smiley-face scale converted into a five-point Likert-type scale for analysis, alongside post-intervention qualitative interviews and focus groups. Semi-structured interviews and focus groups were based on the theoretical framework of acceptability (TFA) [36]. The latter describes acceptability as a multi-faceted construct which reflects the extent to which people delivering or receiving a healthcare intervention consider it to be adequate, based on anticipated or experienced cognitive and emotional responses to the intervention. The audio-recorded focus groups and semi-structured interviews were conducted after the completion of the intervention. Focus groups explored perceived acceptability, experienced benefits and challenges, social and emotional impacts, and views on sustainability of the Join4Joy approach. Focus groups were planned to include both frail and non-frail residents, with attention to gender balance and inclusion of participants from lower socioeconomic backgrounds.

A mixed-method assessment process was applied across both phases. Quantitative assessments were conducted before and after the 12-week intervention by trained researchers, using standardised instruments. Assessments were carried out on-site in the participating NHs, with support from care professionals when needed to facilitate communication or participation. Following programme completion, qualitative data were collected. Semi-structured interviews and audio-recorded focus groups were conducted by trained research staff in NHs. All discussions were conducted in local languages, transcribed verbatim, anonymised, and translated into English for analysis.

### Sample size

As a feasibility study, with a focus on implementation, acceptability and process outcomes, the sample size calculations were pragmatically conducted, where the study targeted ~ 24 participants per country (≈ 72 total) to estimate the primary outcomes. This approach is consistent with recommendations for feasibility studies, which emphasise estimating process outcomes rather than achieving statistical power [37]. No interim analyses or stopping rules were applied. As a non-randomised feasibility study, all participants received the same intervention. Randomisation was not applied, as the primary aim was to deliver an inclusive and adaptable programme accessible to a broad range of residents [38].

### Analysis

#### Quantitative analysis

Quantitative data were analysed in IBM SPSS Statistics v29. Descriptive statistics summarised feasibility and outcome measures. Pre–post changes were explored using paired t tests. Exploratory effect sizes (Cohen’s d) were calculated for pre–post-changes by dividing the mean change by the standard deviation (SD) of the baseline distribution. Reporting effect sizes in feasibility studies is recommended to contextualise the magnitude of change when samples are small and hypothesis testing is not the primary aim [38, 39]. They should be interpreted cautiously as preliminary estimates of potential effect and variability, rather than formal effect sizes.

#### Qualitative analysis

Qualitative data were analysed using reflexive thematic analysis, following Braun and Clarke’s approach [40]. This involved familiarisation with the data, generation of initial codes, and iterative development of themes through a reflexive and interpretative process. Coding was conducted independently by two researchers, followed by discussions to refine themes and ensure coherence and credibility of interpretation. Analysis focused on identifying patterns of meaning across participants’ accounts rather than quantifying responses. In line with reflexive thematic analysis, the emphasis was placed on depth and interpretative richness rather than thematic saturation.

#### Mixed-methods integration

A convergent parallel design was applied, in which quantitative and qualitative data were collected and analysed independently but integrated during interpretation. The combination of mixed methods provided a comprehensive assessment of feasibility and process outcomes. The quantitative data served to evaluate trial protocol feasibility, document missing data patterns, and confirm the viability of the statistical approach for a future definitive trial. Concurrently, the qualitative findings provided an interpretive process evaluation.

## Results

### Participant flow

A total of 81 NH residents participated in the Join4Joy–NH intervention across two phases (Phase 1: *n* = 39; Phase 2: *n* = 42). Six participants withdrew during the intervention period (Phase 1: *n* = 2; Phase 2: *n* = 4), resulting in complete data for 75 participants (Phase 1: *n* = 37; Phase 2: *n* = 38), who were included in the analyses. In Phase 1, three groups were delivered: Germany-P1 (*n* = 11), France-P1 (*n* = 9), and Spain-P1 (*n* = 17). In Phase 2, four groups were delivered: Germany-P2 (*n* = 9), France-P2 (*n* = 10), and two separate Spanish groups (Spain-P2A: *n* = 9; Spain-P2B: *n* = 10). Two Spanish groups in Phase 2 were delivered within the same NH but included different participants and facilitators; therefore, they were reported as distinct groups.

### Sociodemographic and baseline characteristics

The sample across all phases was predominantly female (76.0%), with a mean age of 84–85 years. Cognitive function varied: around 60–70% of participants had no or mild impairment, while the remaining ones showed moderate or higher deterioration according to the Reisberg Global Deterioration Scale. Most residents had achieved primary education only and several (13.3%) required wheelchairs. Prior leisure-time PA was common, with more than 80% reporting previous engagement. Full sociodemographic details are provided in Table 1.

**Table 1:** Sociodemographic data of participants in all the sites

	Total sample (*N* = 75)
Number of females (%)	57 (76.0%)
Mean age, years (SD)	83.7 (10.4)
Mean BMI (SD)	26.1 (6.6)
Number of participants with previous PA experience (%)	65 (86.7%)
Number of participants with moderate or higher cognitive impairment (%)	28 (37.3%)
Number of wheelchair users (%)	10 (13.3%)

*BMI* body mass index, *PA* physical activity, *SD* standard deviation

### Feasibility and acceptability outcomes

Feasibility outcomes included recruitment, attendance, and retention rates, as well as successful intervention delivery, research processes, multicentre working, and data completion. Acceptability was quantitatively assessed by using participant satisfaction ratings. Attendance and retention across all sites were high (87.4% and 92.6%, respectively), with participation remaining stable throughout the 12-week programme. Six participants discontinued the programme, due to relocation (*n* = 3), death (*n* = 2), or acute health events (*n* = 1). None of these incidents were related to participation in the intervention and, therefore, were not classified as adverse events. Satisfaction with the sessions was consistently positive. A detailed summary of feasibility and acceptability outcomes is presented in Table 2.

**Table 2:** Quantitative feasibility and acceptability outcomes, measured by mean satisfaction of the sessions, attendance, and retention in all the sites

	Total sample (*N* = 75)
Mean satisfaction of the sessions (points in the scale 1–5 (SD))	4.3 (0.5)
Mean attendance rate (absolute figure out of 12 sessions (%))	10/12 (87.4%)
Retention rate (*n/N* (%))	75/81 (92.6%)

*SD* standard deviation

### Physical and psychosocial outcomes

Through both phases, participants demonstrated small but generally positive changes. In Phase 1, trends pointed toward modest improvements in physical function, perceived improvement, and PA levels, together with a slight reduction in sedentary time. In Phase 2, improvements were most apparent in daily functioning and PA, while other measures remained relatively stable. Results were pooled for final analysis due to the small, exploratory nature of the sample sizes across individual cohorts. None of the pre–post changes reached statistical significance. Participants also reported perceived improvement and high enjoyment, reflected in favourable PGI-I and PACES-SF scores in all the phases (mean scores of the PACES-SF scale were 4.1 ± 0.7 out of maximum score 5.0 for the total sample, while mean PGI-I scores were 3.8 ± 0.8 out of maximum score 5.0). Effect-size estimates for the outcomes were small across the phases (Cohen’s d generally < 0.30), reflecting very small magnitude of pre-post change. An overview of mean changes (Δ ± SD), *p* values, and effect sizes for all outcomes is presented in Table 3, with full detailed data provided in Annex I (Supplementary Material).

**Table 3:** Mean changes (Δ ± SD), *p* values and effect sizes for quantitative outcomes

	Variable	Δ ± SD	*p* value	Cohen’s *d*
Physical function	SPPB (points)	+ 0.1 ± 3.2	0.5	0.0
Daily functioning	Modified Barthel Index (points)	+ 3.0 ± 28.5	0.4	0.1
Quality of life	EQ-5D-5L index	– 0.1 ± 0.4	0.3	– 0.2
	EQ-5D-5L VAS score	– 1.7 ± 18.5	0.5	– 0.1
Sedentary behaviour	SBQ (hours/week)	– 0.6 ± 20.9	0.9	0.0
Physical activity	IPAQ-SF (MET-min/week)	+ 629.2 ± 2672.2	0.2	0.2

*SD* standard deviation, *SPPB* Short Physical Performance Battery, *EQ-5D-5L* EuroQol 5-dimension 5-level questionnaire, *VAS* visual analogue scale, *SBQ* sedentary behaviour questionnaire, *IPAQ-SF* International Physical Activity Questionnaire-Short Form

### Accelerometry measurements

Accelerometer data were obtained in Spain and Germany, allowing device-based objective assessment of PA and sedentary behaviour across both phases. Valid accelerometry data were available for 37 participants. Analyses are exclusively based on participants with valid accelerometry data. Following the 12-week intervention, objective movement patterns remained largely unchanged among the participants. Minimal, non-significant directional shifts were observed for average daily sedentary minutes and daily light-intensity PA time. Effect sizes for accelerometry-derived variables were very small (Cohen’s *d* < 0.20), indicating negligible shifts in movement behaviour. Summary of the accelerometry results is presented in Table 4, with detailed information available in the Annex I of the Supplementary Material section.

**Table 4:** Mean changes (Δ ± SD), *p* values and effect sizes for accelerometry-derived PA and sedentary time

	Δ ± SD	*p* value	Cohen’s d
% Time in sedentary behaviour	− 0.1 ± 7.5	0.9	0.0
Sedentary time (min/day)	− 2.5 ± 139.1	0.7	0.0
% Time in light PA	− 0.2 ± 7.7	0.9	0.0
Light PA (min/day)	− 3.1 ± 103.8	0.8	0.0

*Δ* mean change from baseline to post-intervention, *PA* physical activity, *SD* standard deviation, *min/day* minutes per day

### Qualitative outcomes

Post-intervention interviews were conducted with all 75 participants to ensure broad inclusion. However, the final thematic framework was built upon the 46 datasets that provided sufficient narrative depth for coding. While individuals with moderate-to-severe cognitive impairment (Reisberg Scale ≥ 3) participated fully in the intervention, their less coherent answers did not yield meaningful codes.

**Fig. 1:**
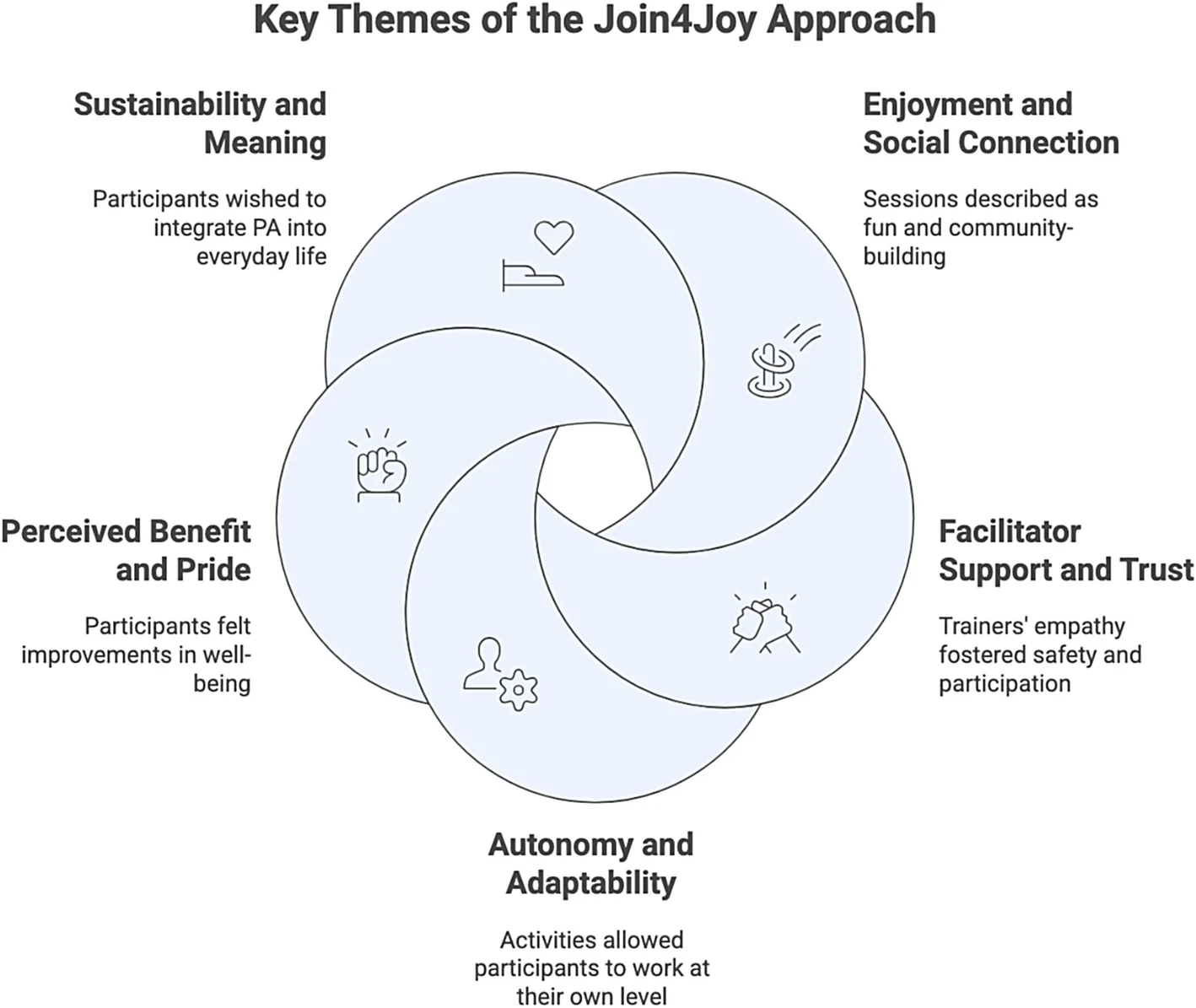
Summary of qualitative findings

In addition, three post-intervention focus groups were conducted following Phase 2: one in Spain (*n* = 12 residents), one in Germany (*n* = 3), and one in France (*n* = 3). Focus groups were moderated by trained researchers and held in familiar settings within the NHs. Thematic analysis of responses from 46 interview participants, together with insights from the focus groups, revealed consistent patterns across countries and groups. Qualitative findings are reported thematically across participants rather than stratified by phase, as focus groups were conducted only after Phase 2 and the analysis aimed to capture shared experiences across implementation contexts. They also focus on shared patterns of experience rather than frequency of responses. Across interviews and focus groups, five interrelated themes emerged that describe the Join4Joy approach: enjoyment and social connection, facilitator support and trust, autonomy and adaptability, perceived benefit and pride, and sustainability and meaning (Fig. 1).

#### Enjoyment and social connection

Most participants described the Join4Joy sessions as enjoyable, socially engaging, and as a meaningful part of their weekly routine. Enjoyment, interaction, and structure were cited as primary motivators across participants. Several residents provided detailed accounts of the positive emotional impact, such as: “Very good, I enjoyed it. I felt better… I feel like I can do things. It makes me happy” (male, Spain).

The positive social atmosphere was frequently highlighted as an important contributor to engagement, captured vividly by one participant: “This gym has helped me get to know people… they like me a lot” (female, Spain).

#### Facilitator support and trust

Facilitator support and trust were widely emphasised across participants. Many residents reported feeling well supported by both trainers and peers, with most participants indicating that they felt adequately supported during the sessions. Collective encouragement and a sense of being cared for were recurring elements, illustrated by the remark: “I do what I can… they help me a lot and encourage me” (male, Spain).

When barriers occurred, participants often noted that facilitators responded appropriately and attentively. For example, one participant explained: “One day I hurt my arm, but they took very good care of me” (female, France).

#### Autonomy and adaptability

Themes of autonomy and adaptability were strongly reflected in participants’ accounts. Many residents reported that sessions were well tailored to their abilities, with participants consistently indicating that the exercises suited their functional capacities. Participants frequently emphasised being able to engage at their own pace and according to their limitations, as reflected in the statement: “Walking more than a few steps was difficult, but two steps were fine” (female, France).

Some participants referred to functional limitations, such as restricted upper-limb mobility (“I couldn’t do anything with my left arm anymore”, male, Germany), while others described fluctuations in mood or motivation (“Some days I didn’t feel like going, but I went anyway”, female, Spain). Despite these challenges, participants consistently emphasised trying their best within their abilities.

Approximately half of the participants actively co-designed small adjustments to the sessions, most related to adding music or increasing variety.

#### Perceived benefit and pride

Participants frequently described perceived improvements in flexibility, mobility, or mood. Participants described increased confidence, particularly in relation to balance and movement, as illustrated by the comment: “Yes… I’m less afraid; I feel more confident with balance” (female, France). Others reflected on perceived longer-term functional benefits, such as: “I can walk a few more steps now… I practice every day because it helps my bones” (female, Germany).

Enjoyment and satisfaction with the programme were high, reported widespread, reinforcing a sense of pride and achievement associated with participation.

#### Sustainability and meaning

Willingness to continue PA after the programme was high, reflecting the sustainability and personal meaning attributed to the Join4Joy experience. Participants expressed varying levels of confidence regarding continued engagement, for example: “As long as the head still works, I will continue” (female, Spain). Others noted that ongoing participation might require external support: “It’s complicated without someone to push me” (female, France).

Some participants described challenges related to fluctuating health, motivation, or memory, such as feeling unwell on certain days (“Some days I didn’t feel like going, but I went anyway”, female, Spain) or difficulty recalling exercises (“I hardly remember… I don’t remember almost any exercise”, female, Spain).

#### Cross-country perspectives

Country-specific nuances were observed but aligned with the shared thematic structure. In Spain, residents often described the programme as nostalgic and emotionally meaningful (“When we danced… music. If I am well, yes, I like it”, female, Spain). In France, participants emphasised confidence and safety (“Yes… except for the balance, I’m less afraid now”, female, France). In Germany, residents particularly valued the structured and supportive environment (“Yes, really enjoyed it… less on health, more on well-being”, female, Germany). Across all sites, participants consistently characterised Join4Joy as safe, enjoyable, socially meaningful, and supportive of ongoing engagement with movement in daily life.

### Integrated findings

Integration of quantitative and qualitative data revealed strong overall coherence across feasibility, biopsychosocial, and experiential outcomes. Retention rates were high (88.0%), and attendance remained consistently good across sites, ranging from 74.0 to 95.0%. No adverse events were reported, and instances of participant withdrawal were primarily due to health-related issues unrelated to the programme.

Quantitative findings showed small and variable changes across outcomes, which should be interpreted cautiously given the exploratory nature of the analyses. Improvements in SPPB, EQ-5D-5L VAS scores, and IPAQ-SF values indicated a general trend toward enhanced PA and quality of life. These findings were supported by the qualitative data, where participants frequently described feeling more flexible, active, and emotionally uplifted following the sessions. Qualitative insights also provided depth to the interpretation of feasibility and acceptability outcomes. Participants consistently expressed enjoyment, appreciation for social connection, and satisfaction with the supportive and adaptable nature of the sessions.

## Discussion

The results of this mixed-methods feasibility study suggest that the Join4Joy approach is feasible, safe, and acceptable for institutionalised older adults. High attendance, retention, and satisfaction across sites, together with the absence of adverse events, support the practicality of implementing this approach within diverse NH contexts. These findings align with the primary feasibility objectives of the study and indicate that Join4Joy can be delivered safely and consistently in real-world long-term care settings.

### Physical and psychosocial outcomes

Although small changes were observed in physical performance, this study was not designed to determine clinically important change, as anchor-based methods, responsiveness indices, and measurement error were not assessed. While minimal clinically important difference (MCID) thresholds for the SPPB of approximately 0.3–0.5 points have been reported in frail and pre-frail populations [28, 41], these values are provided here only as contextual reference points and cannot be directly applied to this NH sample. In this context, the maintenance of lower-limb function and mobility—rather than continued deterioration—may be considered a positive and encouraging outcome within this population. Given the advanced age, high frailty, and limited life expectancy of many NH residents [13, 14], preserving current functional capacity holds substantial practical value for safety, independence, and quality of life [18, 42]. Viewed within this population-specific context, the functional stability and modest improvements observed in this study should, therefore, be interpreted as encouraging and hypothesis-generating findings.

### Exploratory

Analyses indicate that greater adherence and enjoyment may be related to functional and quality-of-life outcomes. These findings align with the previous literature showing that older adults who sustain higher levels of participation in exercise programmes are more likely to achieve favourable functional and psychosocial outcomes [12]. Likewise, systematic reviews confirm that regular PA is consistently associated with improved quality of life in older populations [43]. Descriptive comparisons showed similar patterns of improvement in physical performance and enjoyment across countries, supporting the feasibility of implementing Join4Joy in diverse European care settings. However, given the absence of a control group and the exploratory nature of the analyses, these findings should be interpreted cautiously and cannot be attributed directly to the intervention.

### Objective and self-reported PA outcomes

Self-reported questionnaires, particularly the IPAQ-SF and SBQ, showed considerable variability between participants, which is likely influenced by cognitive status and subjective interpretation of PA and sedentary behaviour. In this study, this variability was reflected in wide inter-individual ranges and small, inconsistent mean changes in self-reported PA and sedentary time, which contrasted with the more stable patterns observed in accelerometry data. This observation is consistent with previous evidence suggesting that cognitive impairment may reduce the accuracy of self-reported PA in older adults, often resulting in over- or underestimation when compared with objective measures [44]. Thus, while self-reported instruments remain valuable for capturing perceived changes and subjective experience, these results reinforce the need for cautious interpretation of questionnaire-based outcomes in institutionalised populations with cognitive decline.

Complementary accelerometry data showed generally stable activity behaviour, with small positive shifts towards reduced sedentary time and increased light-intensity PA. Incomplete wear-time and technical data losses likely increased variability and attenuated detectable changes. These challenges mirror common limitations reported in accelerometry research with older adults, including wear-time compliance, device positioning, and technical difficulties [45, 46].

### Interpretation of mechanisms and expected trajectories

The observed patterns, while modest, are broadly consistent with mechanisms described in the literature on exercise and frailty in ageing populations [47]. The Join4Joy sessions combined low-intensity multicomponent activity with rhythmic and enjoyable movement, potentially enhancing neuromuscular activation, proprioceptive control, and cardiovascular conditioning. Such interventions may contribute to maintaining aspects of physical function such as muscle power, reaction time, and gait stability which are associated with reduced risk of falls and dependency [48]. Systematic reviews have further demonstrated that exercise programmes targeting balance and strength reduce the risk of falls and mobility decline in older adults [49].

The findings of this study suggest that the Join4Joy approach may support functional stability rather than dramatic clinical improvement in a NH population. Given the progressive nature of frailty in long-term care, maintaining existing levels of physical function and daily functioning can be considered a positive outcome [50]. The high levels of enjoyment reported by participants indicate that the social and playful nature of the intervention successfully engaged residents, potentially fostering a more positive affective response to movement [51].

The discrepancy between the perceived improvements reported by residents and the stable objective data from accelerometry suggests that the benefits of the intervention may be primarily psychosocial. The social support and trust built between facilitators and residents appear to be key drivers of this perceived benefit [52]. While the 12-week duration was sufficient to demonstrate feasibility and high acceptability, it may have been an insufficient trajectory to observe measurable shifts in sedentary behavior or chronic physical conditions. Future research should focus on whether sustained engagement in such interventions could potentially contribute to reducing social isolation over time [53]. Importantly, given the low frequency and intensity of the intervention, substantial physiological adaptations would not be expected, and the findings should primarily be interpreted in terms of feasibility and engagement rather than clinical effectiveness.

### Qualitative findings and mixed-methods integration

The intervention’s emphasis on enjoyment and social interaction may have contributed to higher engagement and adherence, consistent with the self-determination theory [50]. Positive affect during movement is known to enhance motivation, adherence, and psychophysiological regulation, suggesting that emotional and social mechanisms may partly mediate the functional benefits observed [51]. Our qualitative findings support these interpretations: participants highlighted playfulness, group cohesion, and social connection as key motivators, describing sessions as joyful, meaningful, and inclusive.

Themes of trust, humour, and integration into daily routines indicated that Join4Joy extended beyond traditional exercise, fostering a sense of belonging and purpose—key determinants of mental well-being in institutionalised older adults [52, 53]. These findings are consistent with evidence showing that co-created and socially engaging PA programmes enhance intrinsic motivation, reduce loneliness, and improve quality of life in long-term care residents [53]. Moreover, the findings suggest that the perceived benefits of the intervention may be driven more by psychosocial mechanisms, such as social connection, emotional engagement, and perceived competence than by measurable physiological change.

### Adaptability, sustainability, and implementation potential

The Join4Joy approach demonstrated strong contextual adaptability, with each site adjusting delivery to local conditions while following some core principles. The inclusion of music, storytelling, and creative movement supported motivation and emotional engagement. Many participants expressed intentions to remain active beyond the programme. Participating NHs also expressed interest in continuing the approach within routine activities, highlighting its perceived feasibility and organisational relevance, although no follow-up data were collected to confirm continued engagement beyond the intervention period.

Such adaptability aligns with evidence showing that tailored interventions accounting for older adults’ preferences, abilities, and environments are more successful in maintaining PA over time [42]. Furthermore, programmes adapted to individual or group needs demonstrate better adherence and longer-term maintenance [54, 55]. Together, these findings suggest that Join4Joy has potential as a sustainable and transferable model for long-term care settings.

### Strengths and limitations

A major strength of this study is its multicentre, international design, providing cross-cultural feasibility insights across three countries with distinct organisational cultures. The mixed-methods design further allowed for a comprehensive evaluation, capturing both measurable and experiential outcomes. Furthermore, the co-design process ensured that the intervention was tailored to the specific needs of the target population [56], enhancing its ecological validity.

However, several limitations must be acknowledged. As a non-randomised feasibility study with a small sample size, the findings should be interpreted as exploratory rather than evidence of effectiveness. The absence of a control group limits causal interpretation, and the sample (which included high proportions of participants with no or mild cognitive impairment and relatively low levels of mobility) may not fully represent the broader NH population. While residents with moderate-to-severe cognitive impairment participated in the intervention, their data were excluded from qualitative thematic analysis to ensure data clarity and reliability. This decision was made to ensure the validity of findings, yet the qualitative findings may underrepresent the perspectives of residents with more advanced cognitive decline, limiting the generalisability of conclusions regarding acceptability and experience across the full NH population. Furthermore, the selection of outcome measures in NH populations presents inherent challenges, including sensitivity to change, floor effects, and variability related to cognitive and functional status. Furthermore, the significant heterogeneity in participant age across different sites (ranging from a mean of 79.1–91.9 years) may have acted as a clinical modifying factor, potentially influencing baseline physical function and the overall responsiveness to the intervention. Additionally, the relatively high proportion of participants with no or mild cognitive impairment and relatively low levels of mobility limitation may reflect a degree of selection bias, potentially limiting the representativeness of the sample compared to the broader NH population. The absence of a control group also limits the ability to distinguish intervention-related changes from natural variability or progression in this population. This is particularly relevant in long-term care settings, where health trajectories are often characterised by gradual decline.

Regarding data collection, accelerometry was unavailable at some sites due to logistical constraints. Where used, missing data reflected challenges common in frail populations, such as participant refusal due to discomfort, unfamiliarity with devices, and inconsistent wear-time. The exclusion of accelerometry diaries and the influence of seasonal weather may have further reduced contextual understanding and contributed to measurement bias. Additionally, self-reported measures may be subject to recall bias, particularly in this population. Finally, monitoring session intensity through facilitator observation rather than a standardised scale may have introduced variability in estimating participant effort, although the high enjoyment scores suggest a strong affective appeal regardless of intensity.

### Implications for practice and future research

Findings suggest that Join4Joy appears to be feasible for integration into routine NH activities with minimal resource requirements; however, confirmation of effectiveness requires adequately powered controlled trials. The feasibility parameters obtained through this study can inform sample size calculations for multicentre RCTs. Crucially, moving toward a definitive trial, researchers should re-evaluate traditional endpoints. Future studies should propose suitable non-traditional outcomes—such as structured measures of enjoyment, meaningful occupation, or social inclusion. In a complex institutionalised population receiving a multi-faceted intervention, focusing solely on a single biomedical primary outcome may fail to capture the true, heterogeneous benefits of a co-created PA approach.

## Conclusions

Delivery of the Join4Joy approach in NHs suggests that an enjoyable, co-created PA intervention is feasible, safe, and perceived as beneficial by NH residents, justifying a future large-scale definitive RCT. While physical performance remained stable, the qualitative insights of the participants emphasise that future definitive trials must look beyond traditional biomedical endpoints. Evaluating holistic psychosocial parameters alongside physical metrics will be essential to fully capture the value of co-created PA approaches in long-term care settings.

## Supplementary Information

Below is the link to the electronic supplementary material.

Supplementary file1 (DOCX 59 KB)
