# Supplementary material for: Feasibility, acceptability, and preliminary effects of the Join4Joy approach for older adults living in nursing homes: a multicentre mixed-methods feasibility study
Source: Eur Geriatr Med. 2026 Aug 31;17(5):3113–25. doi: 10.1007/s41999-026-01547-2 (PMC13755007; doi:10.1007/s41999-026-01547-2)
Supplement: Supplementary file 1 [file 41999_2026_1547_MOESM1_ESM.docx]

**Annex I.** Detailed results of participants in Phase 1 and Phase 2 in all the sites.

**Table 1.** Results of the tests of participants before and after intervention in Phase 1 and Phase 2 in all the sites.

|  | **Phase 1** | | | | | | | | **Phase 2** | | | | | | | | | |
| --- | --- | --- | --- | --- | --- | --- | --- | --- | --- | --- | --- | --- | --- | --- | --- | --- | --- | --- |
|  | **Germany (n=11)** | **Germany (n=11)** | **France(n=9)** | **France (n=9)** | **Spain (n=17)** | **Spain (n=17)** | **Total (n=37)** | **Total (n=37)** | **Germany (n=9)** | **Germany (n=9)** | **France (n=10)** | **France (n=10)** | **Spain A (n=9)** | **Spain A (n=9)** | **Spain B (n=10)** | **Spain B (n=10)** | **Total (n=38)** | **Total (n=38)** |
| **Physical function (SPPB)** | 4.00 (3.44) | 4.36 (3.17) | 4.00 (2.92) | 5.78 (3.07) | 3.76 (3.11) | 3.82 (3.24) | 3.89 (3.08) | 4.46 (3.19) | 2.44 (1.81) | 1.11 (2.42) | 4.00 (4.11) | 3.20 (3.13) | 2.89 (2.57) | 3.33 (3.57) | 4.90 (3.67) | 5.12 (4.03) | 3.60 (3.24) | 3.24 (3.52) |
| **Quality of life (EQ-5D-5L)** | 0.70 (0.33) | 0.64 (0.31) | 0.54 (0.18) | 0.48 (0.26) | 0.32 (0.38) | 0.31 (0.36) | 0.49 (0.36) | 0.45 (0.35) | 0.62 (0.23) | 0.54 (0.29) | 0.28 (0.47) | 0.26 (0.40) | 0.54 (0.23) | 0.27 (0.38) | 0.65 (0.36) | 0.53 (0.40) | 0.52 (0.36) | 0.40 (0.38) |
| **EQ-5D-5L VAS scale** | 61.73 (21.54) | 54.55 (25.44) | 59.44 (14.89) | 70.00 (11.73) | 56.59 (13.45) | 57.53 (12.65) | 58.81 (16.24) | 59.68 (17.84) | 58.33 (19.53) | 39.44 (28.77) | 55.00 (26.46) | 52.00 (17.83) | 53.89 (18.67) | 55.33 (12.74) | 56.70 (12.29) | 59.60 (15.30) | 55.97 (19.18) | 51.81 (20.21) |
| **Sedentary behaviour (SBQ)** | 24.38 (13.03) | 19.02 (15.34) | 35.83 (23.42) | 38.06 (23.78) | 54.74 (10.72) | 55.81 (12.20) | 41.11 (20.01) | 40.55 (22.59) | 31.11 (21.21) | 14.86 (11.19) | 30.23 (21.77) | 38.65 (15.65) | 50.28 (22.11) | 50.92 (15.37) | 42.58 (14.37) | 45.93 (18.47) | 38.44 (20.94) | 37.84 (20.21) |
| **Daily functioning (Modified Barthel Index)** | 70.73 (27.27) | 64.73 (28.65) | 87.89 (10.83) | 89.56 (8.82) | 62.82 (30.29) | 63.76 (30.09) | 71.27 (27.26) | 70.32 (27.75) | 75.67 (38.20) | 64.56 (39.52) | 54.10 (36.46) | 66.20 (31.23) | 72.44 (21.77) | 81.67 (16.70) | 75.80 (23.10) | 84.30 (15.56) | 69.26 (30.94) | 74.24 (27.84) |
| **Physical activity (IPAQ-SF)** | 311.09 (620.74) | 370.91 (479.91) | 3807.39 (4017.64) | 6879.50 (3743.16) | 685.15 (402.36) | 589.68 (420.49) | 1333.41 (2411.20) | 2054.61 (3309.96) | 402.72 (352.36) | 360.56 (481.93) | 2312.70 (3347.32) | 2789.10 (2728.92) | 2671.17 (3085.33) | 3078.17 (3130.45) | 1665.00 (1435.86) | 2910.60 (1659.35) | 1774.79 (2459.03) | 2314.36 (2424.40) |

Colours: blue – pre-intervention data, green – post-intervention data. Tests: SPPB – Short Physical performance Battery, SBQ – Sedentary Behaviour Questionnaire, IPAQ-SF – International Physical Activity Questionnaire-Short Form

Table 2. Results of participants’ improvement and enjoyment after the delivery of the programme in Pilot 1 and Pilot 2 in all of the sites

|  | **Phase 1** | | | | **Phase 2** | | | | |
| --- | --- | --- | --- | --- | --- | --- | --- | --- | --- |
|  | **Germany (n=11)** | **France (n=9)** | **Spain (n=17)** | **Total (n=37)** | **Germany (n=9)** | **France (n=10)** | **Spain A (n=9)** | **Spain B (n=10)** | **Total (n=38)** |
| **Perceived Improvement (PGI-I)** | 3.73 (0.65) | 3.38 (0.97) | 3.71 (1.05) | 3.64 (0.92) | 3.67 (0.50) | 3.90 (0.99) | 4.22 (0.83) | 3.80 (0.42) | 3.90 (0.72) |
| **Enjoyment (PACES-SF)** | 3.85 (0.64) | 4.39 (0.68) | 4.49 (0.58) | 4.28 (0.67) | 4.11 (0.49) | 4.43 (0.53) | 3.50 (0.59) | 3.50 (0.90) | 3.89 (0.74) |

PGI-I – Patient Global Impression of Improvement, PACES-SF – Physical Activity Enjoyement Scale Short Form

Table 3. The accelerometry data of participants in Phase 1 and Phase 2 in all of the JOIN4JOY sites.

|  | **Phase 1** | | | | | | **Phase 2** | | | | | | | |
| --- | --- | --- | --- | --- | --- | --- | --- | --- | --- | --- | --- | --- | --- | --- |
|  | **Germany (n=11)** | **Germany (n=11)** | **Spain (n=9)** | **Spain (n=9)** | **Total (n=20)** | **Total (n=20)** | **Germany (n=8)** | **Germany (n=8)** | **Spain A (n=4)** | **Spain A (n=4)** | **Spain B (n=5)** | **Spain B (n=5)** | **Total (n=17)** | **Total (n=17)** |
| **% of time in Sedentary** | 86.56 (6.59) | 86.48 (6.77) | 85.33 (8.92) | 85.56 (8.00) | 86.01 (7.53) | 86.07 (7.16) | 88.84 (7.16) | 90.22 (6.17) | 91.50 (8.71) | 92.97 (0.98) | 93.83 (8.59) | 89.37 (11.40) | 90.93 (7.75) | 90.62 (7.16) |
| **Average Sedentary Minutes per Day** | 1168.45 (160.35) | 1181.18  (126.56) | 1107.54 (171.51) | 1114.00 (149.57) | 1141.04 (163.97) | 1150.95 (137.93) | 1268.25 (114.30) | 1281.08 (111.41) | 1287.57 (105.28) | 1254.50 (142.47) | 1287.57 (114.05) | 1235.43 (189.86) | 1278.48 (105.57) | 1261.40 (136.62) |
| **% of time in Light PA** | 13.44 (6.59) | 13.53 (6.77) | 14.67 (8.92) | 14.44 (8.00) | 13.99 (7.53) | 13.94 (7.16) | 11.16 (7.16) | 9.78  (6.17) | 8.5 (8.71) | 7.03  (10.98) | 8.50 (8.59) | 10.63 (11.40) | 9.75 (7.55) | 9.38 (8.58) |
| **Average Light PA Minutes per Day** | 180.10 (90.25) | 184.99 (91.29) | 188.86 (112.76) | 187.38 (102.91) | 184.04 (98.29) | 186.07 (94.06) | 158.52 (101.09) | 137.56 (85.09) | 121.64 (124.20) | 96.46  (151.32) | 121.64 (123.77) | 144.00 (148.48) | 139.00 (107.48) | 129.78 (115.51) |

Colours: blue – pre-intervention data, green – post-intervention data

**Annex II.** The TIDieR checklist for the feasibility trial.

The TIDieR (Template for Intervention Description and Replication) Checklist

Information to include when describing an inter ention and the location of the information

m

| **Item Item** | **Where located **** | |
| --- | --- | --- |
| **number** | Primary paper  (page or appendix number) | Other ^†^ (details) |
| **BRIEF NAME**  **1.** Provide the name or a phrase that describes the intervention. | Lines 1-2 |  |
| **WHY** |  |  |
| **2.** Describe any rationale, theory, or goal of the elements essential to the intervention. | Lines 128-132 |  |
| **WHAT** |  |  |
| **3.** Materials: Describe any physical or informational materials used in the intervention, including those | Lines 167-170, 264-265, 272-273 |  |
| provided to participants or used in intervention delivery or in training of intervention providers. |  |  |
| Provide information on where the materials can be accessed (e.g. online appendix, URL). |  |  |
| **4.** Procedures: Describe each of the procedures, activities, and/or processes used in the intervention, | Lines 220-224, 234-240 |  |
| including any enabling or support activities. |  |  |
| **WHO PROVIDED** |  |  |
| **5.** For each category of intervention provider (e.g. psychologist, nursing assistant), describe their | Lines 230-232 |  |
| expertise, background and any specific training given. |  |  |
| **HOW** |  |  |
| **6.** Describe the modes of delivery (e.g. face-to-face or by some other mechanism, such as internet or | Line 216-229 |  |
| telephone) of the intervention and whether it was provided individually or in a group. |  |  |
| **WHERE** |  |  |
| **7.** Describe the type(s) of location(s) where the intervention occurred, including any necessary | Lines 193-203 |  |
| infrastructure or relevant features. |  |  |

| **WHEN and HOW MUCH**   8. Describe the number of times the intervention was delivered and over what period of time including Lines 220-224 the number of sessions, their schedule, and their duration, intensity or dose.   **TAILORING**   9. If the intervention was planned to be personalised, titrated or adapted, then describe what, why, Lines 216-220, when, and how. 227-229   **MODIFICATIONS**   10. **^ǂ^** If the intervention was modified during the course of the study, describe the changes (what, why, Lines 164-165 when, and how).   **HOW WELL**   11. Planned: If intervention adherence or fidelity was assessed, describe how and by whom, and if any Lines 247-254 strategies were used to maintain or improve fidelity, describe them. 12. **^ǂ^** Actual: If intervention adherence or fidelity was assessed, describe the extent to which the Lines 362-370   intervention was delivered as planned. |  |
| --- | --- |

** **Authors** - use N/A if an item is not applicable for the intervention being described. **Reviewers** – use ‘?’ if information about the element is not reported/not sufficiently reported.

† If the information is not provided in the primary paper, give details of where this information is available. This may include locations such as a published protocol or other published papers (provide citation details) or a website (provide the URL).

ǂ If completing the TIDieR checklist for a protocol, these items are not relevant to the protocol and cannot be described until the study is complete.

- We strongly recommend using this checklist in conjunction with the TIDieR guide (see *BMJ* 2014;348:g1687) which contains an explanation and elaboration for each item.
- The focus of TIDieR is on reporting details of the intervention elements (and where relevant, comparison elements) of a study. Other elements and methodological features of studies are covered by other reporting statements and checklists and have not been duplicated as part of the TIDieR checklist. When a **randomised trial** is being reported, the TIDieR checklist should be used in conjunction with the CONSORT statement (see www.consort‐statement.org) as an extension of **Item 5 of the CONSORT 2010 Statement.**

When a **clinical trial protocol** is being reported, the TIDieR checklist should be used in conjunction with the SPIRIT statement as an extension of **Item 11 of the SPIRIT 2013 Statement** (see www.spirit‐statement.org). For alternate study designs, TIDieR can be used in conjunction with the appropriate checklist for that study design (see www.equator‐network.org).
